# The diagnostic value of left atrial stiffness for heart failure with preserved ejection fraction in patients with paroxysmal atrial fibrillation

**DOI:** 10.1186/s44156-026-00107-5

**Published:** 2026-02-23

**Authors:** Jinzhi Lai, Xiaohan Qin, Dingding Zhang, Jiaqi Wang, Jiaqi Yu, Keyue Sun, Deyan Yang, Jingbo Fan, Lihua Zhang, Zhongwei Cheng, Kangan Cheng, Peng Gao, Hua Deng, Ligang Fang, Taibo Chen, Quan Fang, Yongtai Liu

**Affiliations:** 1 Department of Cardiology, Peking Union Medical College Hospital, Chinese Academy of Medical Science and Peking Union Medical College, Beijing, China; 2 Medical Research Center, Peking Union Medical College Hospital, Chinese Academy of Medical Sciences and Peking Union Medical College, Beijing, China

**Keywords:** Left atrial stiffness, Mitral annular early diastolic peak velocity, Heart failure with preserved ejection fraction, Paroxysmal atrial fibrillation

## Abstract

**Aims:**

Left atrial (LA) function is impaired in both atrial fibrillation (AF) and heart failure with preserved ejection fraction (HFpEF). Diagnosing HFpEF remains challenging, especially in AF patients. This study aims to evaluate whether LA stiffness (LAS) can facilitate the diagnosis of HFpEF in patients with paroxysmal atrial fibrillation (pxAF).

**Methods and results:**

A total of 187 pxAF patients were enrolled in the discovery cohort and underwent baseline transthoracic echocardiography (TTE). LAS was measured in all patients, and HFpEF was ascertained based on the H_2_FPEF score (44 high-probability of HFpEF [hp-HFpEF], 143 low-probability of HFpEF [lp-HFpEF]). The left ventricular diastolic function and LA function were significantly altered in the hp-HFpEF group compared to the lp-HFpEF group. Among all echocardiographic indices, the ratio of E/MVE’ to 3D LAEF as a surrogate of LAS showed the greatest diagnostic performance in distinguishing hp-HFpEF from lp-HFpEF in pxAF patients (AUC = 0.862, 95% CI 0.801–0.922, *p* < 0.001), with an optimal cutoff value of 0.27. Combining MVE’ and LAS revealed the highest diagnostic efficiency (LR + 12.18) compared to any single variable. The discriminatory value of LAS with MVE’ was further verified in the validation cohort with an LR + of 7.18.

**Conclusion:**

LAS analyzed by conventional echocardiography combined with MVE’ is a non-invasive, decision-support approach for discriminating hp-HFpEF from lp-HFpEF in pxAF patients. The clinical trial registration number: ClinicalTrials.gov NCT05266144.

**Supplementary Information:**

The online version contains supplementary material available at 10.1186/s44156-026-00107-5.

## Introduction

Atrial fibrillation (AF) is the most common sustained cardiac arrhythmia in adults worldwide [1]. The age-standardized AF prevalence is about 1.6% in Chinese adults [2]. Besides increasing age, other comorbidities such as hypertension, diabetes mellitus, coronary atherosclerotic heart disease, obesity and heart failure (HF) significantly contribute to AF development and progression [1]. Heart failure with preserved ejection fraction (HFpEF) accounts for about 50% of HF cases with an LVEF ≥ 50%, in which AF is more common than in those of heart failure with reduced ejection fraction (HFrEF) [3], where the primary pathophysiological change is impaired left ventricular (LV) diastolic function, often accompanied by LA dysfunction [4, 5]. And increasing burden of AF was related to more severe LA mechanical impairment [6].

AF and HFpEF share several common symptoms such as dyspnea and exercise intolerance. AF and HFpEF often co-exist, leading to worse clinical outcomes than either disorder alone [7]. Symptoms in AF were previously attributed solely to arrhythmia, but restoring sinus rhythm does not always alleviate symptoms, suggesting underlying LA myopathy and undiagnosed HFpEF [5]. AF-HFpEF is characterized by LA remodeling, LA mechanical dysfunction, and poor prognosis without significant LV performance alterations [5]. In HFpEF, consistent exposure to elevated LV filling contributes to increased LA stiffness (LAS), LAS and LA function are progressively impaired, with the degree of LAS and LA dysfunction correlating with AF burden [8, 9].

Currently, diagnosing HFpEF is particularly challenging in AF patients. The most valid diagnosis by hemodynamic measures is invasive and costly. Several echocardiographic parameters have been used to diagnose HFpEF, including LA volume, LV hypertrophy, mitral annular early diastolic peak velocity (MVE’), E/MVE’ ratio, and tricuspid regurgitation velocity [4]. Current guidelines recommend two score-based algorithms (H_2_FPEF and HFA-PEFF), which include several specialized tests, limiting their routine clinical application and the parameters mainly focus on LV diastolic function [10, 11]. Therefore, finding simple and feasible echocardiographic parameters to diagnose HFpEF in AF patients is of great importance.

A previous study indicated that LAS was the most accurate index for discriminating HFpEF from simple diastolic dysfunction [12]. LAS can be assessed by the ratio of change in LA pressure to volume during passive filling of LA. In this study, we aim to evaluate whether LAS, defined as the ratio of E/MVE’ to LA emptying fraction (LAEF), can facilitate the diagnosis of HFpEF in patients with pxAF.

## Methods

### Patients and study design

Consecutive pxAF patients admitted to Peking Union Medical College Hospital from September 1, 2020, to September 30, 2022, were prospectively screened for the discovery cohort. PxAF patients from May 1, 2023, to December 31, 2023, were screened for the validation cohort. Paroxysmal AF diagnosis followed the 2020 ESC guidelines, requiring ECG documentation with an episode lasting at least 30 seconds and terminates spontaneously or with intervention within 7 days of onset [1]. HFpEF was diagnosed using the H_2_FPEF score [11]. The H_2_FPEF score includes the following variables: body mass index, at least two antihypertensive medications, atrial fibrillation, pulmonary artery systolic pressure, age, and Doppler echocardiography E/MVE’. Patients with score of 6–9 were considered as high probability of HFpEF (hp-HFpEF) and placed in the hp-HFpEF group, while others were placed in the low probability of HFpEF (lp-HFpEF) group. All patients underwent blood tests and comprehensive transthoracic echocardiography (TTE) at baseline.

The inclusion criteria included: (1) fulfillment of the diagnostic criteria for pxAF; (2) age between 18 and 80 years; (3) anteroposterior diameter of LA < 50 mm according to TTE; (4) LVEF$\geq$ 50% according to TTE. The exclusion criteria were as follows: (1) valvular AF or significant valvular heart disease confirmed by echocardiography (moderate to severe stenosis or insufficiency); (2) history of hyperthyroidism; (3) history of cardiomyopathy, congenital heart disease, unstable coronary disease or recent revascularization; (4) previous radiofrequency catheter ablation (RFCA).        

The study was approved by the Ethics Committee of Peking Union Medical College Hospital (No. ZS-2494) and was conducted in accordance with the Declaration of Helsinki principles and International Conference on Harmonisation Guideline for Good Clinical Practice. Written informed consent was obtained from all patients.

Baseline information of eligible patients with AF was collected including demographics, clinical parameters (co-morbidities, medications and CHA_2_DS_2_-VASc score), laboratory test indices (N-terminal pro-brain natriuretic peptide [NT-proBNP] and high sensitivity C-reactive protein [hsCRP]), and TTE variables. NT-proBNP was measured using electrochemiluminescence immunoassay with Elecsys instrument and reagent kit (Roche Diagnostics, Indianapolis, IN).

### Echocardiography

Baseline comprehensive TTE of all subjects were performed with an ultrasound system (EPIQ7C, Philips Ultrasound, Netherlands), and echocardiographic recordings were obtained with a 1.6–3.2 MHz transducer following the guidelines of the American Society of Echocardiography [13] by two experienced cardiologists. Offline analysis was conducted using QLAB software (Philips Ultrasound, Netherlands). All echocardiographic measurements for this study were performed during sinus rhythm or following successful cardioversion to sinus rhythm to ensure stable hemodynamics and minimize cycle-to-cycle variability. Doppler-derived parameters and left atrial volumes were measured by averaging five consecutive cardiac cycles. Left atrial strain was analyzed using 2D speckle-tracking on three consecutive cycles.

Left atrial volumes (LAVs) were obtained at maximum (LAV_max_, at end-systole before mitral valve opening) and minimum (LAV_min_, at end-diastole when mitral valve closes) volumes and immediately before atrial systole (before the electrocardiographic P-wave) from 3- dimensional echocardiography (3DE) using commercialized software. According to 3DE rendered minimal and maximal LAVs, left atrial emptying fraction (LAEF) was defined as: LAV_max_-LAV_min_/LAV_max_. Global LA strain curve was analyzed in 4-chamber view of 2-dimensional (2D) image. A representative cardiac cycle was selected, and the LA endocardial border was automatic tracking of acoustic markers throughout the cardiac cycle. LA phasic strain was defined as follows: LA_reservoir strain_ (LASr)= peak longitudinal LA strain at the end of the systole, LA_booster train_ = strain value at the end of diastasis immediately before the onset of its downslope and occurring after the beginning of the P wave on the electrocardiogram, and LA_conduit strain_ = LA_reservoir stran_ – LA_booster strain_.

Left ventricular end-diastolic diameter (LVEDD) and LV end-systolic diameter (LVESD) were obtained in conventional parasternal long-axis view. Left ventricular ejection fraction (LVEF) were measured using the modified biplane Simpson equation in the apical four- and two-chamber views. LV longitudinal strain was measured in the apical four-chamber, apical two-chamber and apical long-axis views by automatic tracing the endocardial border of the left ventricle throughout the representative cardiac cycle. LV global longitudinal strain (GLS) was calculated as the average peak longitudinal strain in three views and used for statistical analysis. LV diastolic parameters measurements included the ratio of mitral inflow at early (E) and late (A) diastolic velocity (E/A), mean of lateral and sepal mitral annular early diastolic peak velocity (MVE’) and E/MVE’. LAS was calculated as E/MVE’ divided by 3D LAEF.

### Sensitivity analysis

Given the significant contribution of AF to the H_2_FPEF score (3 points), we conducted a sensitivity analysis using a re-defined definition of HFpEF at baseline. Only symptomatic patients were included in this analysis. Given the overlap of symptoms of HF and AF, symptoms were defined as either a NYHA functional class or CCS (Canadian Cardiovascular Society) class of II-IV. Echocardiographic criteria are defined as structural abnormalities that fulfill morphological criteria for HFpEF including left atrial dilation (left atrial diameter of > 40 mm or left atrial volume index of > 34 mL/m^2^) and left ventricular hypertrophy by increased ventricular thickness (either septum or posterior wall thickness above the sex specific normal value cutoff). Those meeting both symptomatic and structural criteria based on echocardiography were classified as re-defined HFpEF.

### Reproducibility analysis

The reproducibility of LAS was assessed in 10 randomly selected patients by 2 experienced cardiologists blinded to the clinical diagnosis. Intra- and inter-observer agreement was evaluated using intraclass correlation coefficients (ICCs).

### Statistical analysis

Categorical variables were displayed as numbers (percentage), and continuous variables were expressed as mean ± standard deviation (SD) or median (interquartile range [IQR]) with normal and non-normal distribution which was determined using the Kolmogorov-Smirnov test. Group comparisons were performed using two-tailed t-test or Mann-Whitney U test, and proportions were compared using chi-square test or Fisher exact test. Univariate logistic regression analyses were performed to calculate odds ratios (OR) and define potential factors related to hp-HFpEF. Then multivariate logistic regression analysis with backward stepwise selection was conducted using factors with a statistical value of p < 0.05 in the univariate analyses. Receiver-operating characteristic (ROC) analysis, area under the curve (AUC), sensitivity, specificity, positive predictive value (PPV), negative predictive value (NPV) and likelihood ratio (LR) were used to determine the diagnostic accuracy of hp-HFpEF in pxAF patients from significant echocardiographic variables. The optimal cut-off values of MVE’ and LAS were assessed by the Youden index. Statistical significance was set as a two-tailed P-value < 0.05, and all analyses were performed using SPSS software (version 21.0, SPSS, Inc., IBM).

## Results

### Clinical and echocardiographic characteristics

**Fig. 1:**
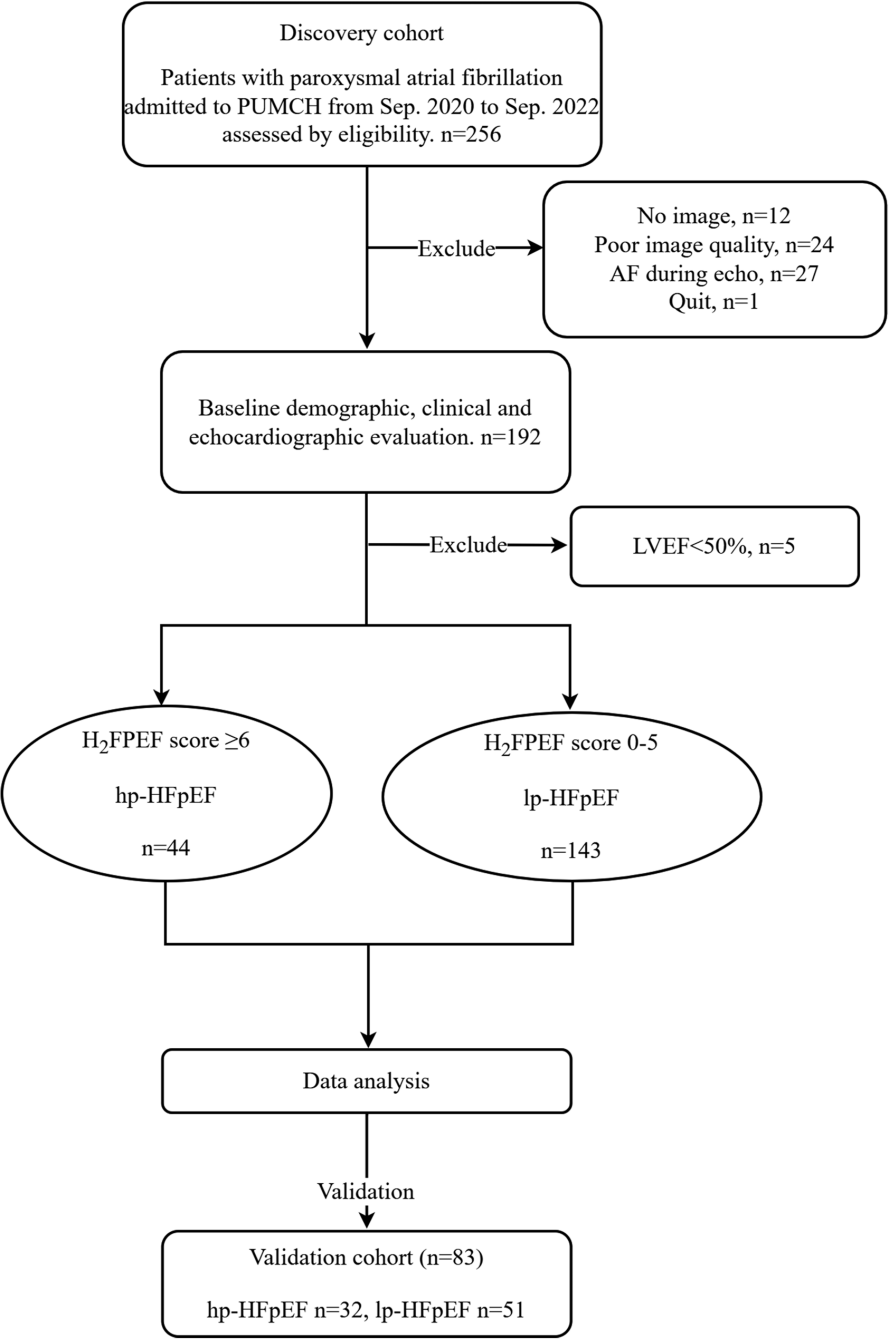
The study workflow. hp-HFpEF, high probability of heart failure with preserved ejection fraction; lp-HFpEF, low probability of heart failure with preserved ejection fraction

A total of 256 consecutive pxAF patients were prospectively screened for the discovery cohort. Among them, 36 subjects lost image or had inadequate image quality, 27 patients were in AF rhythm during TTE, and 1 quit the trial. 192 patients underwent baseline TTE test and LVEF of 5 patients was lower than 50%. Finally, 187 patients (44 hp-HFpEF, 143 lp-HFpEF) were enrolled and analyzed in the discovery cohort. The screening flow chart was shown in Fig. 1. Baseline clinical characteristics and echocardiographic variables were described in Table 1. Patients with hp-HFpEF were older, more obese and more of them were female. Comorbidities such as hypertension, diabetes mellitus, coronary atherosclerotic heart disease or hyperlipidemia were more common in hp-HFpEF group. More patients in hp-HFpEF group had CHA_2_DS_2_-VAS_C_ score $\geq$ 2. NT-proBNP and hsCRP levels were higher in patients with hp-HFpEF.

There was no significant difference in LV mass, dimensions, LVEF and LVGLS between the groups. Hp-HFpEF patients displayed impaired diastolic function with lower MVE’ (6.78 [5.58–7.58] vs. 8.40 [7.35–9.80], *p* < 0.001) and higher E/MVE’ compared to lp-HFpEF patients (12.30 [9.96–14.34] vs. 8.40 [7.35–9.80], *p* < 0.001, Table 1). LAV_min_ and LA anteroposterior diameter were significantly higher, while LAV_max_ showed no significant difference. Variables associated with LA function assessed by LAEF, LASr and LAS were all impaired in hp-HFpEF group. LAEF (49.52±10.00 vs. 56.47±9.58, *p* < 0.001) and LASr (27.4 [21.88–38.33] vs. 33.2 [26.00-41.60], *p* = 0.022) were significantly reduced in the hp-HFpEF compared to the lp-HFpEF group. Meanwhile, LAS, defined as E/MVE’ divided by 3D LAEF, was markedly higher in hp-HFpEF patients compared to lp-HFpEF controls (0.25 [0.21–0.33] vs. 0.15 [0.11–0.19], *p* < 0.001, Table 1).

**Table 1:** Comparison of demographic, clinical and echocardiographic characteristics between hp-HFpEF and lp-HFpEF groups

	hp-HFpEF	lp-HFpEF	*p*-value
Number, *n*	44	143	
**Demographic and clinical variables**			
Age (years)	68 (63–74)	59 (56–68)	< 0.001
Gender, n (%)			0.031
Male	22 (50.0)	98 (68.5)	
Female	22 (50.0)	45 (32.2)	
BMI (kg/m^2^)	27.68 ± 3.63	25.03 ± 2.98	< 0.001
Co-morbidities, n (%)			
Hypertension	41(93.18)	71(49.65)	< 0.001
Diabetes	15(34.09)	19(13.29)	0.003
CHD	12(27.27)	17(11.89)	0.018
Hyperlipidemia	38(86.36)	86(60.14)	0.001
History of stroke	2(4.54)	7(4.90)	0.925
CHA_2_DS_2_-VAS_C_ score, n (%)			< 0.001
0	0	28 (19.58)	
1	5 (11.36)	46 (32.17)	
2	8 (18.18)	37 (25.87)	
3	15 (34.09)	23 (16.08)	
4	11 (25.00)	6 (4.20)	
5	4 (9.09)	3 (2.10)	
6	1 (2.27)	0	
NT-proBNP (ng/L)	158.50(90.50-373.3)	108.0(64.0-215.0)	0.033
hsCRP (mg/L)	1.08(0.63–2.85)	0.80(0.41–1.71)	0.012
Medications, n (%)			
β-blocker	29(65.90)	46(32.17)	< 0.001
ACEI/ARB	35(79.55)	33(23.08)	< 0.001
AADs	12(27.27)	29(20.28)	0.300
Diuretics	5(11.36)	7(4.90)	0.160
**Echocardiographic variables**			
LV mass (mg)	135.0 (117.5–159.0)	135.0 (120.5-165.5)	0.777
LVEDV (mL)	112.0 (93.0-126.0)	119.0 (101.5-142.5)	0.055
LVESV (mL)	38.0 (29.3–53.8)	43.0 (34.5–55.0)	0.137
LVEF (%)	64 (59–70)	64 (59–68)	0.460
LVGLS (%)	18.85 ± 3.02	19.56 ± 2.88	0.202
MVE’ (cm/s)	6.78 (5.58–7.58)	8.40 (7.35–9.80)	< 0.001
E/MVE’ ratio	12.30 (9.96–14.34)	8.28 (6.94–9.84)	< 0.001
LAAPD (mm)	40.02 ± 4.20	38.29 ± 4.58	0.022
LAV_max_ (mL)	69.5 (55.3–94.3)	66.0 (53.0–79.0)	0.140
LAV_min_ (mL)	33.5 (27.3–47.5)	29.0 (21.0–35.0)	0.002
LAEF (%)	49.52 ± 10.00	56.47 ± 9.58	< 0.001
LASr (%)	27.40 (21.88–38.33)	33.20 (26.00-41.60)	0.022
LA stiffness (%^−1^)	0.25 (0.21–0.33)	0.15 (0.11–0.19)	< 0.001

Numerical values are median (interquartile range) or mean ± standard deviation. AF, atrial fibrillation; HF, heart failure; HFpEF, heart failure with preserved ejection fraction; BMI, body mass index; CHD, coronary heart disease; NT-proBNP, N-terminal pro-B-type natriuretic peptide; hsCRP, high sensitivity C-reactive protein; AADs, anti-arrhythmic drugs; LAEF, left atrial emptying fraction; LASr, left atrial reservoir strain; LVEDD, left ventricular end-diastolic diameter; LVESD, left ventricular end-systolic diameter; LVEF, left ventricular ejection fraction; LVGLS, left ventricular global longitudinal strain; MVE’, peak early diastolic mitral annular velocity; LAV, left atrial volume; LAAPD, left atrial anteroposterior diameter

### Related factors of hp-HFpEF in pxAF patients

**Fig. 2:**
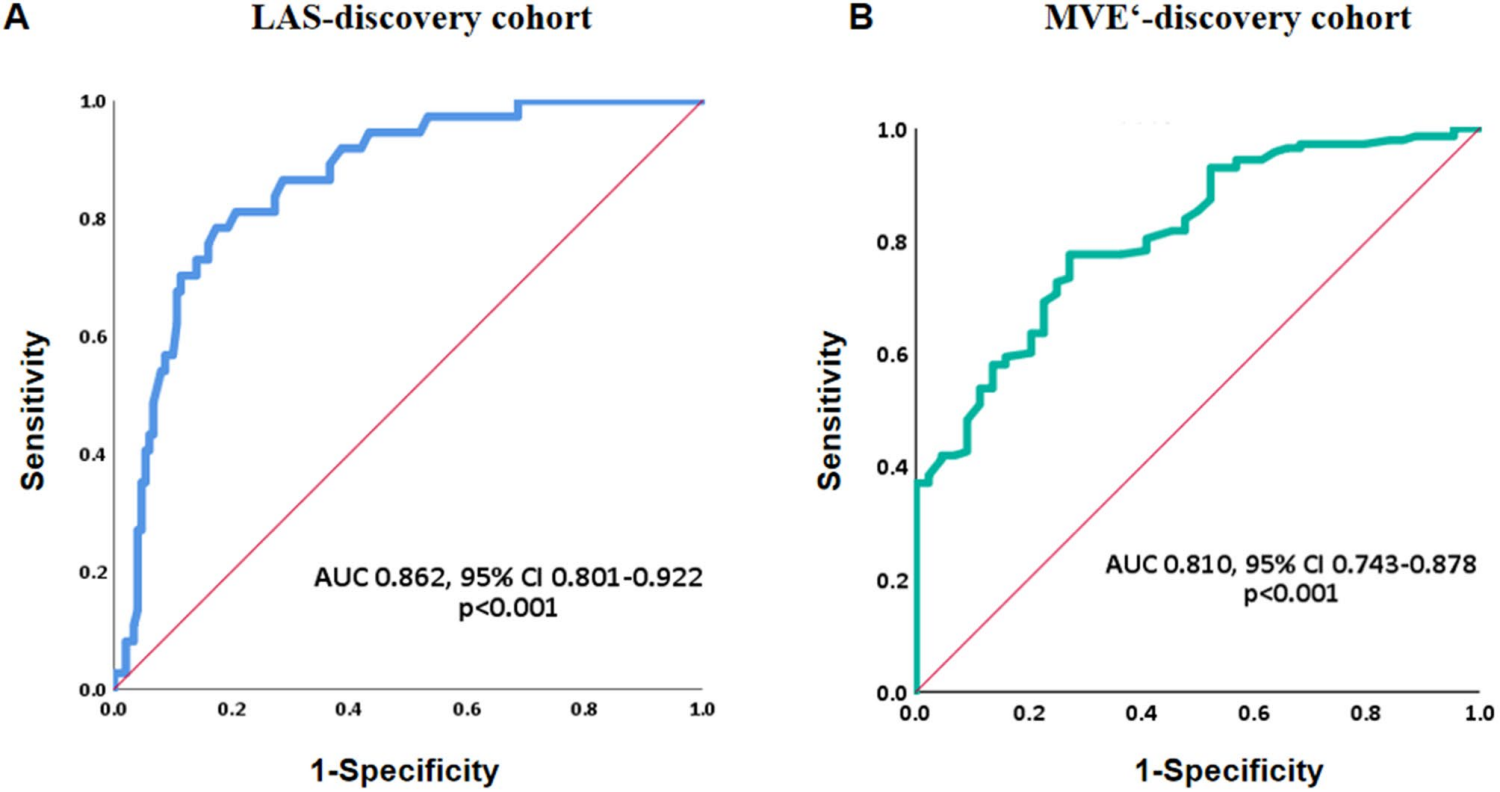
ROC curve showed the performance for (**A**) LAS and (**B**) MVE’ to determine paroxysmal AF patients with hp-HEpEF. LAS, left atrial stiffness; MVE’, mitral annular early diastolic peak velocity

LAS showed good discriminatory performance for HFpEF (AUC = 0.862, 95% CI 0.801–0.922, p < 0.001), with an optimal cut-off value of 0.21 based on the Youden index, providing 77.27% sensitivity and 84.62% specificity (PPV = 60.71%, NPV = 92.37%, LR + = 5.02, LR-=0.27) (Table 2, Fig. 2A). According to the previous research, the normal limit of LAS for elders was set as 0.27 [14]. Thus, we used two cut-off values (0.21 as cut-off value 1, 0.27 as cut-off value 2) for further analyses. We explored the related factors of hp-HFpEF in pxAF patients using univariate and multivariate logistic regression analyses. Variables consisted of the H_2_FPEF score such as age, BMI and comorbidity of hypertension were not included in the logistic regression analyses. LAEF and E/MVE’ which were different between hp-HFpEF and lp-HFpEF group were not included because of their dependency on LAS computational formula. In the univariate logistic analyses, pxAF patients with comorbid hp-HFpEF were associated with gender (OR = 0.459 [0.231–0.914], *p* = 0.027), CHA_2_DS_2_-VAS_C_ score ≤ 1 (OR = 0.120 [0.045–0.321], *p* < 0.001), comorbidity of diabetes (OR = 3.376 [1.534–7.427], *p* = 0.002) or coronary artery disease (OR = 2.779 [1.206–6.404], *p* = 0.016), higher hsCRP (OR = 1.132 [1.002–1.279], *p* = 0.047), LAV index (OR = 1.044 [1.012–1.078], *p* = 0.008), MVE’ (OR = 0.421 [0.308–0.575], *p* < 0.001) and higher LAS (OR = 15.563 [6.938–34.908] for cut-off value 1, OR = 13.451 [5.106–35.432] for cut-off value 2, both *p* < 0.001) (Table 3). We then performed multivariate logistic analyses with backward stepwise selection using variables with *p* < 0.05 in the univariate analyses. Higher MVE’ (OR = 0.577 [0.398–0.836], *p* = 0.004) and fewer LAS ≥ 0.21 (OR = 8.628 [2.837–26.240], *p* < 0.001, model 1) or LAS ≥ 0.27 (OR = 9.144 [2.239–37.338], *p* = 0.002, model 2) were protective factors and had consistent strong association with hp-HFpEF (Table 3). MVE’ demonstrated high discriminatory value for HFpEF (AUC = 0.810, 95% CI 0.743–0.878, *p* < 0.001) with an optimal cut-off value of 7.275, providing 72.70% sensitivity and 77.62% specificity (PPV = 50.00%, NPV = 90.24%, LR + = 3.25, LR-=0.35) (Table 2; Fig. 2B). CHA_2_DS_2_-VAS_C_ score had several common factors with H_2_FPEF score, so we did not take it into further consideration.

### Combined discriminatory performance to AF-hp-HFpEF

Combining the two echocardiographic variables, patients with both MVE’ $\leq$ 7.275 cm/s and LAS $\geq$ 0.27 showed the highest LR+ as 12.18, which was better than any single variable. Patients with both MVE’ $\:\le\:$ 7.275 cm/s and LAS $\geq$ 0.21 also showed high LR + as 7.68 (Table 2). Cutoff value as 0.27 for LAS had better discriminatory performance.    

**Table 2:** Receiver operating characteristic analysis and diagnostic accuracy of MVE’ and LAS to hp-HFpEF in patients with pxAF

Echocardiographic variables	Sen (%)	Spe (%)	PPV (%)		NPV (%)	LR+	LR-
Discovery cohort							
MVE’ ≤ 7.275 cm/s	72.70	77.62	50.00	90.24		3.25	0.35
LAS ≥ 0.21%^−1^	77.27	84.62	60.71	92.37		5.02	0.27
LAS ≥ 0.27%^−1^	40.90	95.10	72.00	84.00		8.35	0.62
MVE’ ≤ 7.275 cm/s and LAS ≥ 0.21%^−1^	59.10	92.30	70.30	88.00		7.68	0.44
MVE’ ≤ 7.275 cm/s and LAS ≥ 0.27%^−1^	34.10	97.20	78.90	82.70		12.18	0.68

HFpEF, heart failure with preserved ejection fraction; pxAF, paroxysmal atrial fibrillation; MVE’, peak early diastolic mitral annular velocity; LAS, left atrial stiffness; PPV, positive predictive value; NPV, negative predictive value; LR, likelihood ratio

**Table 3:** Relationship between clinical parameters and hp-HFpEF: results of univariate and multivariate logistic analysis

Variables	Univariate		Multivariate-Model 1		Multivariate-Model 2	
	OR (95% CI)	*P* value	OR (95% CI)	*P* value	OR (95% CI)	*P* value
gender (male, n %)	0.459 (0.231–0.914)	0.027*	1.170 (0.423–3.238)	0.762	1.245 (0.460–3.370)	0.666
CHA_2_DS_2_-VAS_C_ score ≤ 1	0.120 (0.045–0.321)	< 0.001*	0.234 (0.062–0.879)	0.032	0.199 (0.052–0.757)	0.018
lnNT-proBNP (ng/L)	5.143 (0.950-27.831)	0.057				
LVGLS (%)	0.919 (0.816–1.036)	0.167				
LVEF (%)	1.075 (0.998–1.157)	0.055				
LVMi, g/m^2^	1.001 (0.977–1.024)	0.963				
MVE’ (cm/s)	0.421 (0.308–0.575)	< 0.001*	**0.577 (0.398–0.836)**	0.004*	**0.522 (0.369–0.740)**	< 0.001*
LASr (%)	0.968 (0.937–1.001)	0.054				
hsCRP (mg/L)	1.132 (1.002–1.279)	0.047*	1.068 (0.958–1.191)	0.234	1.071 (0.959–1.197)	0.225
LAVi, mL/m^2^	1.044 (1.012–1.078)	0.008*	0.962 (0.904–1.024)	0.2225	0.967 (0.905–1.032)	0.312
LAS (%^−1^)	3.934 (2.367–6.539)	< 0.001*				
LAS ≥ 0.27	13.451 (5.106–35.432)	< 0.001*			**9.144 (2.239–37.338)**	0.002*
LAS ≥ 0.21	15.563 (6.938–34.908)	< 0.001*	**8.628 (2.837–26.240)**	< 0.001*		
DM, n%	3.376 (1.534–7.427)	0.002*	1.205 (0.404–3.591)	0.7738	1.556 (0.541–4.475)	0.412
CAD, n%	2.779 (1.206–6.404)	0.016*	1.218 (0.355–4.172)	0.754	1.107 (0.344–3.566)	0.865
stroke, n%	0.925 (0.185–4.624)	0.925				
AAD, n%	0.657 (0.301–1.435)	0.292				

Variables with *P* < 0.05 in univariate logistic regression analysis were included in constructing multivariate models. Variables that were significantly associated with hp-HFpEF were indicated. LAS was subjected to model 1 and 2 with cutoff value of 0.21 and 0.27, respectively. NT-proBNP, N-terminal pro- B-type natriuretic peptide; LVGLS, left ventricular global longitudinal strain; LVEF, left ventricular ejection fraction; LVMi, left ventricular mass index; MVE’, peak early diastolic mitral annular velocity; LASr, left atrial reservoir strain; hsCRP, high sensitivity C-reactive protein; LAVi, left atrial volume index; LAS, left atrial stiffness; DM, diabetes mellitus; CAD, coronary artery disease; AAD, anti-arrhythmia drug. *P < 0.05

### Sensitivity analysis of LAS on HF status according to re-defined HFpEF

Of the all patients enrolled in the discovery cohort, 23 patients were symptomatic, high NT-proBNP level (> 125pg/mL) and had echocardiographic data allowing to determine structural presence of HFpEF. In this subgroup, LAS still showed good discriminatory performance for re-defined HFpEF (AUC = 0.859, 95% CI 0.774–0.944, p < 0.001, Fig. S1). Patients with both MVE’ ≤ 7.275 cm/s and LAS ≥ 0.27 still showed high LR + as 10.69.

### Reproducibility of LAS in the discovery cohort

The LAS was repeatedly measured in 10 randomly selected patients, and the intra-observer and inter-observer ICCs were calculated as 0.885 (95%CI 0.624–0.970, *p* < 0.001) and 0.891 (95%CI 0.618–0.972, *p* < 0.001), respectively.

### Discriminatory value of LAS and MVE’ in the validation cohort

A total of 83 patients (32 hp-HFpEF, 51 lp-HFpEF) were enrolled and analyzed in the validation cohort. The baseline characteristics between AF-hp-HFpEF and AF-lp-HFpEF in the validation group were shown in Supplementary Table 1. Similar to the discovery cohort, LAEF, LA volume and LAS were all impaired in hp-HFpEF group. LAS was markedly higher in hp-HFpEF patients compared to lp-HFpEF controls (0.28 ± 0.11 vs. 0.19 ± 0.11, p = 0.001, Supplementary Table 1) and also showed good discriminatory performance for hp-HFpEF (AUC = 0.765, 95% CI 0.664–0.867, p < 0.001, Supplementary Fig. 1). Combining LAS and MVE’ showed high LR + as 7.18 with high specificity of 96.08%, but the sensitivity of the combined factors was poor in the validation cohort (Supplementary Table 2).

## Discussion

In this study, we demonstrated that LAS evaluated by echocardiography is a valuable non-invasive parameter to differentiate hp-HFpEF from lp-HFpEF in pxAF patients. LAS, especially when combined with MVE’, showed significant diagnostic efficiency, providing better discrimination than any single echocardiographic variable.

AF and HFpEF often coexist, leading to increased morbidity and mortality due to shared pathophysiological levels [15]. Each disorder may sequentially lead to the other, or they might have common mechanistic substrate, leading to the parallel manifestation and progression [15]. Shared pathophysiologic mechanisms in AF and HFpEF include system inflammation, neurohormonal activation, upregulation of RAAS, endothelial dysfunction and chronotropic incompetence [16]. Initially, HFpEF was considered LV diastolic dysfunction, but atrial dysfunction was later found to be an important component [4]. AF-HFpEF might be a distinct phenotype characterized by LA dysfunction leading to poor clinical outcomes [17]. Chronically elevated LV filling pressures secondary to AF could cause marked LA dilation and increased LAS in HFpEF [9, 18].

LASr and stiffness were major indicator of LA myopathy, and progressively declined in HFpEF with increasing AF burden [8]. Previous studies have demonstrated that LA strain, especially reservoir strain was associated with poor exercise tolerance and clinical outcome in HFpEF patients [19, 20]. Likewise, when LAS increased, the function as a reservoir chamber of LA was limited, leading to elevated LA pressure [18]. Increased LAS was independently associated with impaired exercise capacity and quality of life [21]. It also correlated to poor clinical outcomes, including increased all-cause mortality and HF hospitalization, and its prognostic role was more prominent than left ventricular filling pressure [12]. But the prognostic value of LAS in AF-HFpEF required further exploration.

Diagnosing HFpEF, especially in AF patients, remains challenging. Previous studies identified HFpEF in AF patients by direct LA pressure measurements [22, 23]. Noninvasive methods have been proposed, particularly echocardiography, to evaluate LV and LA function. Echocardiography is a common tool to assess LA function through LA volume, volumetric change (LAEF) and deformation (strain), etc [24]. Prior studies have indicated that LAS had greater diagnostic value than LASr to differentiate HFpEF patients from those without HF [4, 25]. A previous clinical study showed the E/MVE’ ratio in conjunction with LASr was accurate in identifying diastolic HF patients [25]. In our study, we used E/MVE’ divided by 3D LAEF as the surrogate to LAS. According to the EACVI NORRE study, the limit of normality for LAS in the elder (Age ≥ 60) is 0.27, which showed better diagnostic performance than the cutoff value of Youden index in discovery cohort. Combining LAS, representing LA function, and MVE’, reflecting LV diastolic function, the diagnostic LR+ could reach up to 12.18 in the discovery cohort and 7.18 in the validation cohort. While the AUC in the validation cohort indicates good but not perfect discrimination, the high PPV and LR + of LAS combined with MVE’ operationalize a ‘rule-in’ strategy. This performance profile strategically positions LAS not as a solitary screening tool, but as a high-specificity tool to support the initiation of guideline-directed therapy in patients with paroxysmal AF.

There were several limitations in our study. First, this is a single-center, cross-sectional study which may have referral and selection bias and preclude determination of causality. The performance difference may reflect inherent clinical heterogeneity between the cohorts—a common challenge when validating biomarkers in real-world AF populations. Second, HFpEF was primarily diagnosed using the H_2_FPEF algorithm rather than the gold standard of invasive exercise catheterization, and some patients with HFpEF may be mistaken. Third, LAS is calculated using E/e’, while E/e’ > 9 is a component of the H_2_FPEF score we used as the reference standard. This overlap could theoretically overestimate the diagnostic performance of LAS, but combing LAEF could add independent physiological information. Fourth, our study excludes those with LA diameter ≥ 50 mm which limits the application in AF patients with severe LA remodeling. Next, due to the restriction of LA functional variable accuracy measured by echocardiography in sinus rhythm, our study only included patients with pxAF, which may limit the enrollment of high-risk patients and weaken the generalizability of results.

## Conclusions

In conclusion, our study indicated that patients with hp-HFpEF exhibited severe LA functional impairment. LAS analyzed by echocardiography provided significant value as a noninvasive and high-confidence approach for differentiating hp-HFpEF from lp-HFpEF in patients with pxAF. Furthermore, the combination of LAS and MVE’ offered superior diagnostic efficiency than any single variable.

## Supplementary Information

Below is the link to the electronic supplementary material.

Supplementary Material 1

## Data Availability

The data that support the findings of this study are available from the corresponding author upon reasonable request.
