# Supplementary material for: The diagnostic value of left atrial stiffness for heart failure with preserved ejection fraction in patients with paroxysmal atrial fibrillation
Source: Echo Res Pract. 2026 Feb 23;13:6. doi: 10.1186/s44156-026-00107-5 (PMC12927242; doi:10.1186/s44156-026-00107-5)
Supplement: Supplementary file 1 — Supplementary Material 1 [file 44156_2026_107_MOESM1_ESM.docx]

***Supplementary Materials***

| **Supplementary Table 1. Comparison of demographic, clinical and echocardiographic characteristics between hp-HFpEF and lp-HFpEF in the validation group.** | | | |
| --- | --- | --- | --- |
|  | **hp-HFpEF** | **lp-HFpEF** | **p-value** |
| Number, n | 32 | 51 |  |
| ***Demographic and clinical variables*** |  |  |  |
| Age (years) | 66 (63-73) | 64(55-69) | 0.015 |
| Gender, n (%) |  |  | 0.007 |
| Male | 12(37.5) | 35(68.63) |  |
| Female | 20(62.5) | 16(31.37) |  |
| BMI (kg/m^2^) | 25.34 (23.44-31.10) | 24.57 (22.94-26.47) | 0.037 |
| Co-morbidities, n (%) |  |  |  |
| Hypertension | 29(90.63) | 20(39.22) | <0.001 |
| Diabetes | 11(34.38) | 8(15.69) | 0.062 |
| Coronary heart disease | 4(12.5) | 15(29.41) | 0.107 |
| Hyperlipidemia | 25(78.12) | 40(78.43) | 0.774 |
| History of stroke | 2(6.25) | 2(3.92) | 0.631 |
| CHA_2_DS_2_-VAS_C_ score, n (%) |  |  | 0.012 |
| 0-1 | 5(15.63) | 21(41.18) |  |
| ≥2 | 27(84.37) | 30(58.82) |  |
| lnNT-proBNP (ng/L) | 5.73±1.12 | 4.96±1.04 | 0.002 |
| hsCRP (mg/L) | 1.12(0.50-2.42) | 0.55(0.50-1.09) | 0.031 |
| ***Echocardiographic variables*** |  |  |  |
| LV mass (mg) | 143.72±38.72 | 137.29±27.99 | 0.384 |
| LVEF (%) | 63.58±7.96 | 63.25±5.66 | 0.898 |
| LVGLS (%) | 17.76±3.71 | 17.29±4.75 | 0.693 |
| MVE' (cm/s) | 7.60 (6.99-8.63) | 8.05 (6.90-10.00) | 0.147 |
| E/MVE' ratio | 10.78±2.21 | 8.93±2.65 | 0.001 |
| LAV_max_ (mL) | 84.28±30.82 | 71.92±21.87 | 0.036 |
| LAV_min_ (mL) | 49.38±24.90 | 35.69±17.51 | 0.004 |
| LAEF (%) | 43.20±13.17 | 51.86±14.39 | 0.007 |
| LA reservoir strain (%) | 30.16±8.76 | 30.99±7.88 | 0.798 |
| LA stiffness (%^-1^) | 0.28±0.11 | 0.19±0.11 | 0.001 |
| AF, atrial fibrillation; HF, heart failure; HFpEF, heart failure with preserved ejection fraction; BMI, body mass index; NT-proBNP, N-terminal pro-B-type natriuretic peptide; hsCRP, high sensitivity C-reactive protein; AADs, anti-arrhythmic drugs; LAEF, left atrial empting fraction; LVEDD, left ventricular end-diastolic diameter; LVESD, left ventricular end-systolic diameter; LVEF, left ventricular ejection fraction; LVGLS, left ventricular global longitudinal strain; MVE’, mitral annular early diastolic peak velocity; LAV, left atrial volume; LAAPD, left atrial anteroposterior diameter. | | | |

| **Supplementary Table 2.  Receiver operating characteristic analysis and diagnostic accuracy of MVE' and LAS to pxAF-hp-HFpEF in the validation cohort.** | | | | | | |
| --- | --- | --- | --- | --- | --- | --- |
| **Echocardiographic variables** | **Sensitivity (%)** | **Specificity (%)** | **PPV (%)** | **NPV (%)** | **LR+** | **LR-** |
| MVE' ≤ 7.275 cm/s | 43.75 | 66.67 | 45.16 | 65.38 | 1.31 | 0.844 |
| LAS ≥0.27 %^-1^ | 43.75 | 86.27 | 66.67 | 70.97 | 3.19 | 0.652 |
| MVE' ≤ 7.275 cm/s and LAS ≥0.27 %^-1^ | 28.13 | 96.08 | 81.82 | 63.64 | 7.18 | 0.748 |
| LAS ≥0.21 %^-1^ | 59.38 | 76.47 | 61.29 | 75.00 | 2.52 | 0.53 |
| MVE' ≤ 7.275 cm/s and LAS ≥0.21 %^-1^ | 28.12 | 92.16 | 69.23 | 67.14 | 3.59 | 0.78 |
| HFpEF, heart failure with preserved ejection fraction; pxAF, paroxysmal atrial fibrillation; MVE’, mitral annular early diastolic peak velocity; LAS, left atrial stiffness; PPV, positive predictive value; NPV, negative predictive value; LR, likelihood ratio. | | | | | | |


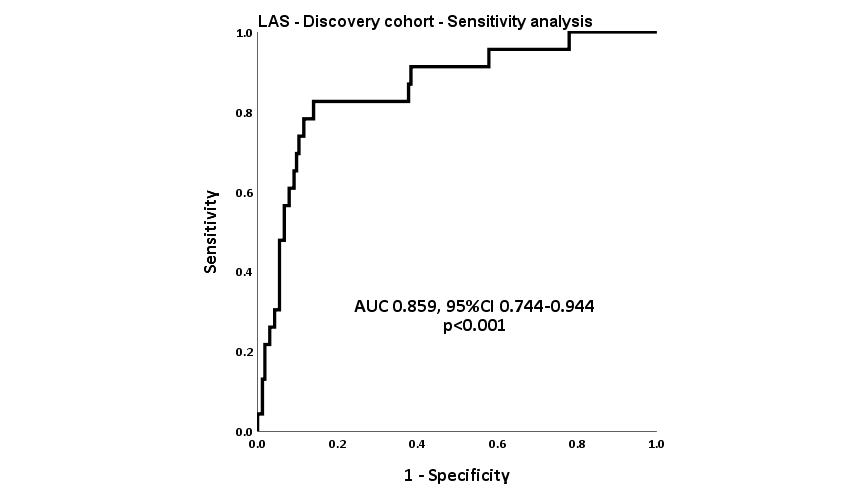


**Supplementary Figure 1**. Receiver operating characteristic curve of left atrial stiffness for discriminating echocardiography-defined HFpEF in the discovery cohort. HFpEF, heart failure with preserved ejection fraction; AUC, area under curve; CI, confidence interval.


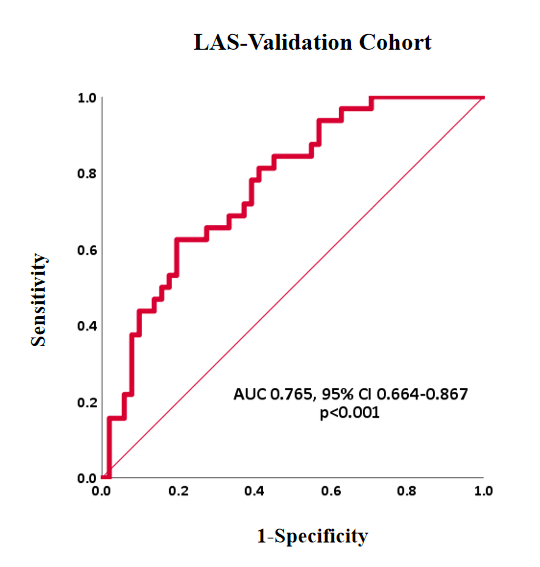


**Supplementary Figure 2.** **Receiver operating characteristic curve of LAS for discriminating hp-HFpEF from lp-HFpEF in the validation cohort.** LAS, left atrial stiffness; HFpEF, heart failure with preserved ejection fraction; AUC, area under curve; CI, confidence interval.
